# JAK inhibitors in autoimmune ocular inflammatory diseases – A systematic review of case reports and series

**DOI:** 10.1186/s12348-025-00537-y

**Published:** 2025-12-10

**Authors:** Salem Almerri, Hamad Alshatti, Yousef Taleb Ali, Raed Behbehani

**Affiliations:** 1 Department of Ophthalmology, Farwaniya Hospital, Kuwait City, Kuwait; 2 Albahar Eye Center, Ibn Sina Hosptial, Kuwait City, Kuwait

**Keywords:** Uveitis, Scleritis, Keratitis, JAK inhibitors, Janus kinase inhibitor, Ocular inflammation, Autoimmune ocular disease

## Abstract

**Background:**

Autoimmune inflammatory ocular diseases, including uveitis, scleritis, and ocular mucous membrane pemphigoid, can cause severe, vision-threatening complications and are often associated with systemic autoimmune conditions. Standard therapies involve corticosteroids, conventional disease-modifying antirheumatic drugs (cDMARDs), and biologics, yet some cases remain refractory. Janus kinase (JAK) inhibitors have emerged as a promising therapeutic option for such refractory cases and are increasingly being explored as potential primary or secondary line therapies.

**Main body:**

This systematic review synthesized evidence from case reports and case series describing the use of JAK inhibitors in non-infectious ocular inflammation. A comprehensive search of PubMed and MEDLINE (from inception to March 2025) identified 22 studies (12 case reports, 10 case series) involving 43 patients (64 eyes). Patients ranged from 13 to 85 years (mean = 41) with a female predominance (69.7%). Reported ocular conditions included scleritis (46.5%), uveitis (34.9%), ocular mucous membrane pemphigoid (7.0%), keratitis (4.7%), keratoconjunctivitis (4.7%), and non-specific orbital inflammation (2.3%). Most patients had failed prior therapies with corticosteroids, cDMARDs (80%), and biologics (51%) before initiating JAK inhibitors. Tofacitinib (62.8%) was the most frequently used agent.

The median follow-up was approximately 2.5 months (range: 1–78 months). Complete remission was achieved in 69.7% of patients, while 27.9% experienced partial or marked improvement. More than half of the patients were able to taper or discontinue corticosteroids. No relapses were reported during follow-up. Reported adverse events were mild and included elevated transaminases, leukopenia, neutropenia, and herpes virus reactivation.

**Conclusion:**

JAK inhibitors demonstrate promising efficacy and an acceptable safety profile in refractory autoimmune ocular inflammatory diseases, enabling high rates of remission and reduced steroid dependence. These findings highlight their potential role in treatment algorithms and underscore the need for further prospective studies to define their long-term efficacy, safety, and optimal use.

## Introduction

Autoimmune inflammatory ocular diseases may occur either in isolation or in association with systemic autoimmune conditions such as rheumatoid arthritis, Sjögren’s syndrome, seronegative spondyloarthropathies, and antineutrophil cytoplasmic antibody (ANCA)-associated vasculitides. Ocular involvement can be in the form of keratitis, scleritis, uveitis, and retinitis [1]. 

Management of autoimmune inflammatory ocular diseases typically follows a stepwise approach, with first line therapeutic agents including nonsteroidal anti-inflammatory drugs (NSAIDs) and/or corticosteroids in various formulations, depending on the severity and extent of inflammation. Second line therapeutic agents include conventional disease-modifying antirheumatic drugs (cDMARDs) such as anti-metabolites like methotrexate, mycophenolate mofetil, and azathioprine. Finally, biologic agents such as adalimumab, infliximab, and golimumab are used in resistant cases to enhance disease control and minimize long-term corticosteroid exposure [2, 3]. 

Janus kinase (JAK) inhibitors have recently emerged as potential therapeutic agents for autoimmune ocular diseases refractory to all three lines of management, and being explored as potential primary and secondary line therapy [4]. JAKs are intracellular tyrosine kinases, comprising several isoforms including JAK1, JAK2, and JAK3, that play a critical role in the JAK/STAT signaling pathway. Upon cytokine receptor activation, JAKs phosphorylate and activate signal transducer and activator of transcription (STAT) proteins, which then translocate to the nucleus to induce pro-inflammatory gene expression. By inhibiting this pathway, JAK inhibitors aim to reduce cytokine-mediated inflammation [5, 6]. 

While JAK inhibitors are already approved for various autoimmune and inflammatory conditions such as rheumatoid arthritis and Crohn’s disease, their application in autoimmune ocular diseases is still limited but has gained increasing attention in recent years [5]. This systematic review aims to summarize the available literature—specifically case reports and case series—on the use of JAK inhibitors in the management of autoimmune inflammatory ocular diseases.

## Methods

This review was conducted according to Preferred Reporting Items for Systematic Reviews and Meta-Analyses guidelines (PRISMA). It is registered on PROSPERO (ID: 1050235).

### Search strategy and study selection

A comprehensive literature search was conducted by two independent investigators using the MEDLINE and PubMed databases to identify relevant articles published up to March 2025. The search strategy involved a combination of keywords related to Janus kinase inhibitors and ocular inflammatory conditions. First, the following terms were combined using the Boolean operator “OR”: “Janus kinase inhibitor”, “JAK inhibitor”, “tofacitinib”, “baricitinib”, “jakinib”, “ruxolitinib”, “filgotinib”, “upadacitinib”, and “brepocitinib”.

Separately, the following search terms were used combined using “OR”: “ocular inflammation”, “episcleritis”, “scleritis”, “uveitis”, “keratitis”, “conjunctivitis”, “retinal vasculitis”, and “retinitis”. The two groups of search terms were then combined using the Boolean operator “AND”. All retrieved articles were screened independently after removing duplication by the two investigators (SM, HS) by using systematic review software Rayyan (Rayyan Cambridge, MA, USA). Any disagreements regarding inclusion were resolved through discussion with a third reviewer (YT).

### Eligibility criteria

We included studies that met the following eligibility criteria: case reports or case series involving patients with a primary diagnosis of non-infectious ocular inflammation, in which Janus kinase (JAK) inhibitors were used specifically for the control of ocular inflammation. Included studies were also required to report follow-up data, including outcomes such as remission status or the degree of clinical improvement. Studies were excluded if they employed a different study design, such as cohort studies, cross-sectional studies, or randomized controlled trials; if the ocular inflammation was attributed to infectious, post-surgical, or traumatic causes; if JAK inhibitors were used for a different primary diagnosis; if follow-up assessments were insufficient; or if the full text of the article was not available after multiple attempts of retrieval.

### Data extraction

Data extraction was performed independently by three reviewers (SM, YT, HS) using a standardized template developed in Microsoft Excel (Microsoft Corp., Redmond, WA, USA). From each included study, the following information was extracted: study characteristics (first author, year of publication, country of origin, study design, and sample size), patient characteristics (age, sex, underlying diagnosis, primary ocular presentation, disease duration, and prior treatments), intervention details (the JAK inhibitor used, dosage, route of administration, duration of treatment, and any concomitant therapies). Outcome data were also collected, including reported remission rates, degree of corticosteroid tapering, recurrence rates, and any documented adverse effects of JAK inhibitors. Since this review is descriptive in nature and based on case reports and case series, a quantitative meta-analysis was not feasible. Instead, the findings were synthesized narratively and presented in detailed summary tables. Relevant clinical data were organized and discussed extensively in the Results section. Where applicable, descriptive statistics such as mean, median, and range were calculated for patient demographics, including age and gender, to provide a clearer overview of the included population.

### Quality assessment

The methodological quality of the included case reports and case series was assessed using the tool proposed by Murad and colleagues, which is specifically designed for critical appraisal of such study designs [7]. This tool evaluates eight items grouped into four key domains: selection, ascertainment, causality, and reporting. Given that the review is based entirely on case reports and case series, the overall certainty of evidence is inherently low, which should be considered when interpreting the findings.

## Results

**Fig. 1:**
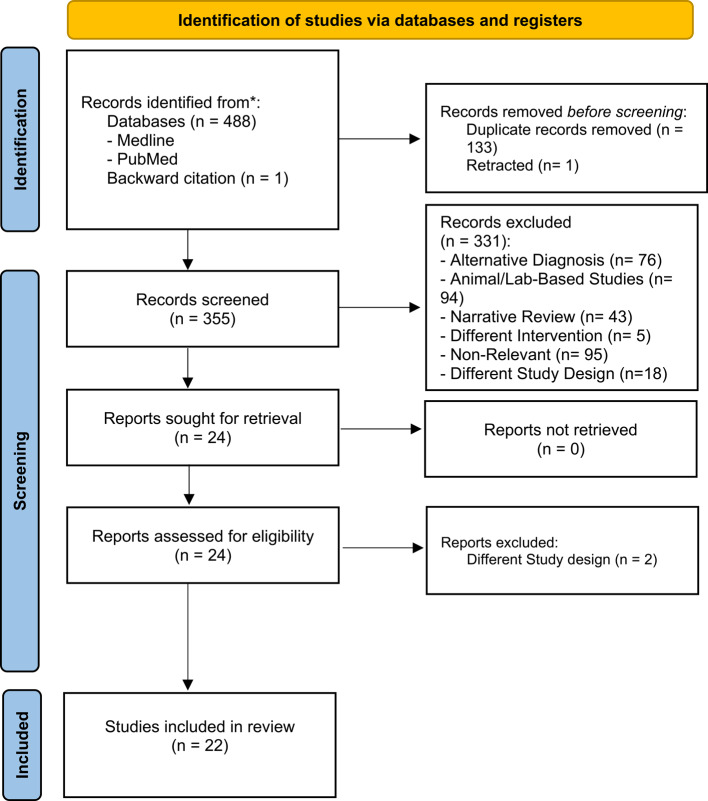
PRISMA flow chart

A comprehensive search of PubMed and MEDLINE databases identified 489 records. After removing 133 duplicates and one retracted article, 355 studies remained for title and abstract screening. Of these, 331 studies were excluded for the following reasons: non-relevant subject matter (*n* = 95), animal or laboratory-based studies (*n* = 94), alternative diagnoses (*n* = 76), narrative reviews (*n* = 43), incompatible study design (*n* = 18), and incompatible intervention (*n* = 5). Twenty-four full-text articles were retrieved for detailed assessment, with two subsequently excluded for not meeting the eligibility criteria (both retrospective cohort studies). Ultimately, 22 studies comprising 43 patients were included in this systematic review. The study selection process is illustrated in Fig. 1.

The included studies consisted of 12 case reports and 10 case series, published between 2014 and 2025. A total of 43 patients (64 eyes) were included, with a mean age of 41 years (range: 13–85), with a female predominance (69.7%). The ocular inflammatory conditions treated with JAK inhibitors included scleritis (46.5%, *n* = 20), uveitis (34.9%, *n* = 15), ocular mucous membrane pemphigoid (7.0%, *n* = 3), keratitis (4.7%, *n* = 2), keratoconjunctivitis (4.7%, *n* = 2), and non-specific orbital inflammation (2.3%, *n* = 1). Associated systemic diseases were reported in 44.2% of patients, most commonly rheumatoid arthritis (11.6%, *n* = 5) and juvenile idiopathic arthritis (11.6%, *n* = 5), followed by Sjögren’s syndrome, systemic lupus erythematosus, granulomatosis with polyangiitis, Behçet’s disease, Vogt-Koyanagi-Harada disease, and psoriasis (Tables 1 and 2).

**Table 1:** Study design characteristics of the included reports, specifying publication type (case report or case series), year of publication, country of origin, and number of patients described

Author	Country	Year	Study Design	Size Sample	Eye Involved	Systemic Disease	JAK inhibitor involved
Vidic Krhlikar ^[8]^	Slovenia	2024	Case Series	2 [Pt 1–2]	Scleritis Non-Specific Orbital Inflammation	-	Baricitinib, Tofacitinib
Chung Young Kim ^[9]^	South Korea	2025	Case Series	3 [Pt 3–5]	Scleritis	RA, SLE	Tofacitinib
Vidic Krhlikar^[10]^	Slovenia	2025	Case Series	3 [Pt 6–8]	Uveitis	-	Baricitinib, Upadacitinib
Baquet-Walscheid^[11]^	Germany	2022	Case Report	1 [Pt 9]	Scleritis	Spondyloarthritis, IgA nephritis	Upadacitinib
Pyare^[12]^	India	2024	Case Series	10 [Pt 10–19]	Scleritis	1 = Sjogern’s Syndrome 1 = GPA	Tofacitinib
Kito^[13]^	Japan	2024	Case Report	1 [Pt 20]	Vernal Keratoconjunctivitis	Atopic Dermatitis	Upadacitinib
Mima^[14]^	Japan	2024	Case Report	1 [Pt 21]	Vernal Keratoconjunctivitis	Atopic Dermatitis	Upadacitinib
Yang^[15]^	China	2024	Case Report	1 [Pt 22]	Tattoo granuloma with uveitis (TAGU)	-	Abrocitinib
Dey^[16]^	India	2023	Case Series	3 [Pt 23–25]	Scleritis	-	Tofacitinib
Tao^[17]^	China	2023	Case Series	2 [Pt 26–27]	Uveitis	Behcet’s Disease	Upadacitinib
Calvo-Río^[18]^	Spain	2022	Case Report	1 [Pt 28]	Keratitis	RA	Baricitinib
Baquet-Walscheid^[19]^	Germany	2022	Case Report	1 [Pt 29]	Uveitis	JIA	Tofacitinib, Upadacitinib
James^[20]^	United States	2022	Case Series	2 [Pt 30–31]	Ocular Mucous Membranous Pemphigoid	-	Tofacitinib
Kaneko^[21]^	Japan	2021	Case Report	1 [Pt 32]	Uveitis	RA	
Liu^[22]^	China	2021	Case Report	1 [Pt 33]	Uveitis	-	Tofacitinib
Pyare^[23]^	India	2020	Case Report	1 [Pt 34]	Scleritis	-	Tofacitinib
Majumder^[24]^	India	2020	Case Report	1 [Pt 35]	Uveitis	VKH	Tofacitinib
Miserocchi ^[25]^	Italy	2020	Case Series	4 [Pt 36–38]	Uveitis	JIA	Tofacitinib, Baricitinib
Bauermann^[26]^	Germany	2019	Case Report	1 [Pt 39]	Uveitis	JIA	Tofacitinib
Paley^[27]^	United States	2019	Case Series	2 [Pt 40–41]	1 – Uveitis 1 – Scleritis	-	Tofacitinib
Meadow^[28]^	United Stated	2014	Case Report	1 [Pt 42]	Keratitis	RA	Tofacitinib
Sarny^[29]^	Austria	2018	Case Report	1 [Pt 43]	Mucous Membrane Pemphigoid	Psoriasis	Baricitinib

*JIA* Juvenile Idiopathic Arthritis, *RA* Rheumatoid Arthritis, *SLE* Systemic lupus erythematosus, *VKH* Vogt-Kayanagi-Harada Disease, *GPA* Granulomatosis with Polyangiitis

**Table 2:** – Patient demographics and prior therapies, including conventional DMARDs and biologic agents, used before initiation of JAK inhibitor therapy

					DMARDs	
Patient	Age	Gender	Ocular Inflammation	Systematic Diseases	Conventional DMARDs	Biological DMARDs
1	46	M	Scleritis	N/A	MTX, MMF, TCR	RTX
2	28	F	Non-Specific Orbital Inflammation	N/A	N/A	N/A
3	38	F	Scleritis	Rheumatoid Arthritis	MTX, CTX, CsA	N/A
4	54	F	Scleritis	Rheumatoid Arthritis	TCR	ABA
5	61	F	Scleritis	SLE	HCQ	N/A
6	28	F	Uveitis	N/A	MMF, TCR	ADA
7	41	M	Uveitis	N/A	MTX, MMF, CsA	ADA
8	59	M	Uveitis	N/A	MMF	ADA
9	42	F	Scleritis	Spondyloarthritis, IgA nephritis	MTX, AZA, CsA	ADA, IFX, GOL
10	47	F	Scleritis	GPA	CTX, MMF	N/A
11	51	F	Scleritis	N/A	MMF	N/A
12	41	M	Scleritis	N/A	AZA	N/A
13	41	F	Scleritis	N/A	N/A	N/A
14	58	F	Scleritis	N/A	N/A	N/A
15	39	F	Scleritis	N/A	MTX	N/A
16	51	M	Scleritis	N/A	MTX, MMF	N/A
17	35	F	Scleritis	Sjogern’s Syndrome	N/A	N/A
18	22	F	Scleritis	N/A	MTX, MMF, AZA	N/A
19	41	M	Scleritis	N/A	N/A	N/A
20	13	F	Vernal Keratoconjunctivitis	Atopic Dermatitis	CsA, TCR	N/A
21	18	F	Vernal Keratoconjunctivitis	Atopic Dermatitis	N/A	N/A
22	41	M	Tattoo granuloma with uveitis (TAGU)	N/A	N/A	N/A
23	53	F	Scleritis & Keratitis	N/A	MMF	ADA
24	18	M	Scleritis	N/A	MTX, MMF	N/A
25	47	F	Scleritis	N/A	MTX	ADA
26	Adolescent	F	Uveitis	Behcet’s Disease	MTX, MMF, CsA	ADA
27	Thirties	M	Uveitis	Behcet’s Disease	MTX, MMF, CsA	ADA
28	85	F	Peripheral Ulcerative Keratitis	RA	N/A	N/A
29	24	F	Uveitis	JIA	MTX, AZA, CsA	ADA, IFX, TCZ
30	79	F	Ocular Mucous Membranous Pemphigoid	N/A	MTX, MMF, CTX	RTX
31	70	M	Ocular Mucous Membranous Pemphigoid	N/A	MTX, MMF, CTX, CsA	RTX
32	35	M	Uveitits	Seronegative Rheumatoid Arthritis	MTX, SZA	ADA, IFX
33	18	F	Uveitis	N/A	MTX, MMF, CsA	ADA
34	65	F	Scleritis	N/A	MMF	N/A
35	24	F	Uveitis	VKH	N/A	N/A
36	18	F	Uveitis	JIA	MTX	ADA, IFX, RTX, ABA
37	37	F	Uveitis	JIA	MTX, AZA	ADA, IFX, GOL, TCZ
38	21	M	Uveitis	JIA	MTX, CsA	ADA, IFX, RTX, ABA, TCZ
39	22	F	Uveitis	JIA	MTX, MMF, CsA	ADA, IFX, RTX, GOL, TCZ
40	45	F	Uveitis	Undefined	MTX, LEF, AZA, MMF	ADA, CER
41	40	F	Scleritis	N/A	MTX, MMF, AZA, CTX	N/A
42	59	F	Ulcerative Keratitis	Rheumatoid Arthritis	MTX	ABA
43	43	M	Mucous Membrane Pemphigoid	Psoriasis	MTX, MMF, CTX	ADA, RTX

*MTX  Methotrexate, *MMF* Mycophenolate mofetil, *TCR* Tacrolimus, *CTX* Cyclophosphamide, *CsA* Cyclosporine A, *HCQ* Hydroxychloroquine, *AZA* Azathioprine, *SZA* Sulfasalazine, *LEF* Leflunomide, *ADA* Adalimumab, *IFX* Infliximab, *GOL* Golimumab, *ABA* Abatacept, *RTX* Rituximab, *TCZ* Tocilizumab, *CER* Certolizumab pegol, *JIA* Juvenile Idiopathic Arthritis, *RA* Rheumatoid Arthritis, *SLE* Systemic lupus erythematosus, *VKH* Vogt-Kayanagi-Harada Disease, *GPA* Granulomatosis with Polyangiitis

The duration of disease prior to JAK inhibitor initiation ranged from immediate treatment to 34 years (mean: 62.5 months). Corticosteroids were used in all but two patients with ocular mucous membrane pemphigoid. Approximately 80% of patients had previously failed conventional DMARDs, most commonly methotrexate, followed by mycophenolate mofetil, azathioprine, cyclophosphamide, and cyclosporine. Biologic agents were used in 51% of patients, including anti-TNF agents (adalimumab, infliximab), anti-CD20 (rituximab), IL-6 inhibitors (tocilizumab), and CTLA4-Ig (abatacept). A total of nine cases where JAK inhibitors were initiated without a trial of cDMARDs or bDMARDs, mostly patients with scleritis. (Table 2)

The JAK inhibitors administered were tofacitinib (62.8%), baricitinib (23.3%), upadacitinib (11.6%), and abrocitinib (2.3%). Two patients with uveitis were switched to upadacitinib from tofacitinib or baricitinib due to poor clinical response. Most patients received tofacitinib at 5 mg twice daily, baricitinib at 4 mg once daily, and upadacitinib at 15 mg once daily, with one patient escalated to 30 mg daily for severe atopic dermatitis. (Table 3)

Complete clinical remission was achieved in 69.7% (*n* = 30) of patients, while partial improvement was observed in 27.9% (*n* = 12). One patient discontinued tofacitinib following the development of herpetic stromal keratitis; therefore, treatment efficacy could not be evaluated in this case. The time to remission ranged from 1 week to 9 months (median: ~2.5 months), with no preference of certain JAK inhibitor. The follow-up duration ranged from 1 to 78 months (mean: ~6.5 years), with 42 patients continuing JAK inhibitor therapy at last follow-up. Notably, none of these patients were able to discontinue JAK inhibitors despite achieving remission. No relapses were reported during follow-up, including the one patient who discontinued tofacitinib. Additionally, at the last follow-up, 32.5% of patients (*n* = 14) were on combination therapy with DMARDs, predominantly methotrexate. Two patients required valacyclovir due to herpes virus reactivation, and in one of these cases, patient was solely on antiviral medication after tofacitinib discontinuation. (Table 3)

**Table 3:** Summary of JAK inhibitor regimen, dosage, treatment duration, concomitant therapies (excluding corticosteroids), and reported adverse events for each patient

					JAK Inhibitors					
Patient	**Age**	**Gender**	**Ocular Inflammation**	**Systematic Diseases**		**JAK Inhibitor**	**Dose**	**Treatment Duration**	**Final Combination** **(Excluding CS)**	**Adverse Effect**
1	46	M	Scleritis	N/A		Baricitinib	4 mg OD	18 months	No	Elevated Transaminase
2	28	F	Non-Specific Orbital Inflammation	N/A		Tofacitinib	5 mg BD	N/A	No	No
3	38	F	Scleritis	Rheumatoid Arthritis		Tofacitinib	5 mg BD	> 2 years	No	No
4	54	F	Scleritis	Rheumatoid Arthritis		Tofacitinib	5 mg BD	> 2 years	No	No
5	61	F	Scleritis	SLE		Tofacitinib	5 mg BD	2 years	No	No
6	28	F	Uveitis	N/A		Baricitinib	4 mg OD	> 23 months	No	Elevated Transaminase
7	41	M	Uveitis	N/A		Baricitinib > Upadacitinib	BAR (4 mg OD) > UPA (15 mg OD)	1 year BAR, then 3 yrs 6mths UPA	No	No
8	59	M	Uveitis	N/A		Baricitinib	4 mg OD	> 17 months	No	No
9	42	F	Scleritis	Spondyloarthritis, IgA nephritis		Upadacitinib	15 mg OD	13 months	No	No
10	47	F	Scleritis	GPA		Tofacitinib	5 mg BD	11 months	No	No
11	51	F	Scleritis	N/A		Tofacitinib	5 mg BD	5 months	No	No
12	41	M	Scleritis	N/A		Tofacitinib	5 mg BD	3 months	No	No
13	41	F	Scleritis	N/A		Tofacitinib	5 mg BD	1 month	Valacivir 500 mg BD	Herpatic stromal keratitiis
14	58	F	Scleritis	N/A		Tofacitinib	5 mg BD	7 months	No	No
15	39	F	Scleritis	N/A		Tofacitinib	5 mg BD	7 months	Leflunomide 10 mg OD	No
16	51	M	Scleritis	N/A		Tofacitinib	5 mg BD	7 months	No	No
17	35	F	Scleritis	Sjogern’s Syndrome		Tofacitinib	5 mg BD	8 months	No	No
18	22	F	Scleritis	N/A		Tofacitinib	5 mg BD	14 months	Valacivir 500 mg BD	Reactivation of Herpetic Labialis
19	41	M	Scleritis	N/A		Tofacitinib	5 mg BD	6 months	No	No
20	13	F	Vernal Keratoconjunctivitis	Atopic Dermatitis		Upadacitinib	15 mg OD > 30 mg OD (Due to AD)	14 months	Antiallergic eye drops	Not Reported
21	18	F	Vernal Keratoconjunctivitis	Atopic Dermatitis		Upadacitinib	15 mg OD	5 months	No	No
22	41	M	Tattoo granuloma with uveitis (TAGU)	N/A		Abrocitinib	100 mg OD	6 months	No	No
23	53	F	Scleritis & Keratitis	N/A		Tofacitinib	5 mg BD	> 6 months	No	N/A
24	18	M	Scleritis	N/A		Tofacitinib	5 mg BD	>3months	MTX 15 mg/wk	N/A
25	47	F	Scleritis	N/A		Tofacitinib	5 mg BD	6 months	MTX 15 mg/wk	N/A
26	Adolescent	F	Uveitis	Behcet’s Disease		Upadacitinib	15 mg OD	11 months	No	Mild Leucopenia and Transiminitis
27	Thirties	M	Uveitis	Behcet’s Disease		Upadacitinib	15 mg OD	9 months	MTX 15 mg/wk, MMF 1 g BD	No
28	85	F	Peripheral Ulcerative Keratitis	RA		Baricitinib	2 mg OD	6 months	No	No
29	24	F	Uveitis	JIA		Tofacitinib > Upadacitinib	15 mg OD	4 months	No	No
30	79	F	Ocular Mucous Membranous Pemphigoid	N/A		Tofacitinib	11 mg OD	14 months	No	No
31	70	M	Ocular Mucous Membranous Pemphigoid	N/A		Tofacitinib	11 mg OD	12 months	MMF 1 g BD	No
32	35	M	Uveitits	Seronegative Rheumatoid Arthritis		Baricitinib	8 mg OD	31 months	No	No
33	18	F	Uveitis	N/A		Tofacitinib	5 mg BD	10 months	MTX 10 mg/wk	No
34	65	F	Scleritis	N/A		Tofacitinib	5 mg BD	> 1 month	MMF 500 mg BD	No
35	24	F	Uveitis	VKH		Tofacitinib	10 mg/day	N/A	No	N/A
36	18	F	Uveitis	JIA		Baricitinib	4 mg OD	7 months	MTX 15 mg/wk	No
37	37	F	Uveitis	JIA		Baricitinib	4 mg OD	5 months	No	No
38	21	M	Uveitis	JIA		Baricitinib	4 mg OD	13 months	MTX 15 mg/wk	No
39	22	F	Uveitis	JIA		Tofacitinib	5 mg BD	4 months	MTX 2.5 mg/wk, FA 5 mg BD	N/A
40	45	F	Uveitis	N/A		Tofacitinib	11 mg OD	9 months	MTX	N/A
41	40	F	Scleritis	N/A		Tofacitinib	11 mg OD	3 months	MTX	N/A
42	59	F	Ulcerative Keratitis	Rheumatoid Arthritis		Tofacitinib	5 mg BD	9 months	No	N/A
43	43	M	Mucous Membrane Pemphigoid	Psoriasis		Baricitinib	4 mg OD	1 month	MTX 25 mg/wk	Low Neutrophil granulocytes

*JIA Juvenile Idiopathic Arthritis, *RA* Rheumatoid Arthritis, *SLE* Systemic lupus erythematosus, *VKH* Vogt-Kayanagi-Harada Disease, *GPA* Granulomatosis with Polyangiitis, *AD* Atopic Dermatitis

*N/A* Not Available, *OD* once a day, BD twice a day

*BAR* Baricitinib, *UPA* Upadacitinib, *MTX* Methotrexate, *MMF* Mycophenolate mofetil

*N/A* Not Available

Steroid tapering or discontinuation was a key measured clinical outcome, given the risk of steroid-related complications. Cataracts were observed in 27.9% (*n* = 12) of patients, and glaucoma in three, likely due to prolonged steroid use. Following JAK inhibitor initiation, 17 patients were able to completely discontinue systemic steroids, while another 11 significantly reduced their dosage. (Table 4)

**Table 4:** Reported symptoms and clinical presentations prior to initiation of JAK inhibitor therapy, disease duration before JAK inhibitor initiation, time to remission following JAK inhibitor treatment, follow-up duration, and reported relapse rates

					Presentation and Outcome						
Patient	Age	Gender	Ocular Inflammation	Systematic Diseases		Clinical Presentation		Disease Duration prior to JAK-I	Remission Time since JAK-I initiation	Follow-Up Duration	Relapses?
1	46	M	Scleritis	N/A		**Left eye** – Redness and pain. Dilated scleral and episcleral vessels, episcleral nodule temporally. **Cataract**		15 years	Complete = 3 weeks	18 months	No
2	28	F	Non-Specific Orbital Inflammation	N/A		**Left eye** – Redness and chemosis. Upper eyelid edema and pain during ocular movement specially on Left gaze. U/S confirmed Medial rectus myositis.		4 months	Improved = 2 months	14 months	No
3	38	F	Scleritis	Rheumatoid Arthritis		**Right Eye** – Refractory, Diffuse congestion of deep and superficial episcleral vascular plexus, scleral edema and tenderness in right eye		2 years	Complete = 8 months	78 months	No
4	54	F	Scleritis	Rheumatoid Arthritis		**Right Eye** – Refractory Anterior Scleritis, Distortion and tortuosity of superficial and deep episcleral vessels, mostly inferior and temporal quadrants with tenderness		15 weeks	Complete = 9 months	48 months	No
5	61	F	Scleritis	SLE		**Bilateral** – Refractory Anterior scleritis and Red eye and pain, Diffuse episcleral congestion and scleral edema		> 3 months	Complete = 8 months	63 months	No
6	28	F	Uveitis	N/A		**Bilateral** – Anterior and intermediate uveitis: Blurred vision with 3 + aqueous and vitreous cells, cystoid macular edema OD, **cataract**		1 year 4 mth	Complete = 4 months	23 months	No
7	41	M	Uveitis	N/A		**Bilateral** – Anterior and intermediate uveitis, diminished visual acuity, Significant vitritis and cystoid macular edema OD, **Glaucoma**,** Cataract**		6 yrs 5 mths	Complete = 6 months	43 months	No
8	59	M	Uveitis	N/A		**Bilateral** – Posterior Uveitis (ampiginous choroiditis), Decreased visual acuity and Increased floaters, granulomatous endothelial precipitates, aqueous and vitreous cells, hyperemic discs, yellowish retinal lesions, pigmented atrophic lesions.		37 months	Complete = 9 months	17 months	No
9	42	F	Scleritis	Spondyloarthritis, IgA nephritis		**Bilateral** - Diffuse anterior and posterior non-necrotizing scleritis		10 years	Complete = 1 week	13 months	No
10	47	F	Scleritis	GPA		**Left Eye** – Necrotizing Diffuse scleritis, scleral thinning and necrotising in superior part of left eye		47 months	Complete = 1 month	11 months	No
11	51	F	Scleritis	N/A		**Left Eye** - Necrotizing Anterior scleritis, severe pain and redness, diffuse congestion of the sclera with peripheral corneal infiltrates, scleral thinning		23 months	Complete = 6 months	5 months	No
12	41	M	Scleritis	N/A		**Right Eye** - Diffuse anterior scleritis		4 months	Improved = 3 months	3 months	No
13	41	F	Scleritis	N/A		**Bilateral** - Diffuse anterior scleritis, Redness and pain, deeper episcleral vessels were diffusely congested, and minimal scleral oedema.		60 months	Discontinued	7 months	No
14	58	F	Scleritis	N/A		**Bilateral** - Diffuse non- necrotizing anterior scleritis		1.5 months	Complete = 6 months	7 months	No
15	39	F	Scleritis	N/A		**Left Eye** - Nodular anterior scleritis, Severe tenderness and pain, diffuse anterior scleritis – inferotemporal nodule.		4.5 months	Complete = 1 month	7 months	No
16	51	M	Scleritis	N/A		**Left Eye** – Nodular anterior scleritis, well-defined nodular scleral elevation with congestion around it and adjacent areas of minimal scleral thinning		19.5 months	Complete = 1 month	7 months	No
17	35	F	Scleritis	Sjogern’s Syndrome		**Left Eye** – Nodular anterior scleritis		0 months	Complete = 2 month	8 months	No
18	22	F	Scleritis	N/A		**Right Eye** – Nodular anterior scleritis, Inferior scleral congestion, with a solitary nodular lesion associated with no tenderness, **Glaucoma**		26 months	Complete = 8 months	14 months	No
19	41	M	Scleritis	N/A		**Left Eye** – Active diffuse anterior & posterior scleritis, raised intraocular pressure and sub-retinal fluid at the posterior pole		1.5 months	Improved = 6 months	6 months	No
20	13	F	Vernal Keratoconjunctivitis	Atopic Dermatitis		**Right Eye** – Photophobia, itchy eyes, and pain. Giant papillae on upper tarsal conjunctiva and SPKs		48 months	Complete = 3 months	14 months	No
21	18	F	Vernal Keratoconjunctivitis	Atopic Dermatitis		**Bilateral** - Giant cobblestone papillae and thickened tarsal conjunctivae, accompanied by a mucous discharge. Severe peripheral corneal vascularization, diffuse punctate keratitis, residual paracentral leukomas were observed		0 months	Complete = 2 month	5 months	No
22	41	M	Tattoo granuloma with uveitis (TAGU)	N/A		**Bilateral** – Blurred vision, lacrimation, photophobia		5 months	Complete = 5 weeks	6 months	No
23	53	F	Scleritis & Keratitis	N/A		**Right Eye** – pain, redness, photophobia, and decreased vision. Scleral necrotizing nodule and intense hyperaemia were observed, and sclera thinning – necrotising + keratitis temporally		12 months	Complete = 6 months	6 months	No
24	18	M	Scleritis	N/A		**Left Eye** – pain, redness, and discomfort upon ocular movements. Anterior nodular scleritis in the nasal quadrant		N/A	Complete = 2 week	3 months	No
25	47	F	Scleritis	N/A		**Left Eye** – Diffuse Anterior Scleritis, pain and redness		11 months	Complete = 6 months	6 months	No
26	Adolescent	F	Uveitis	Behcet’s Disease		**Bilateral –** Panuveitis, Cystoid Macular Edema, Retinitis, Vasculitis		6 months	Complete = 6 months (inc. CME)	11 months	No
27	Thirties	M	Uveitis	Behcet’s Disease		**Right Eye –** Blurred vision. Panuveitis, and Cystoid macular Edema		21 months	Complete = 6 months	9 months	No
28	85	F	Peripheral Ulcerative Keratitis	RA		**Right Eye** - Epithelial defect with stromal thinning, led to extensive perforation near the limbus. Subsequently sutured prior to use of JAK-I		1 year	Clinically improved = 2 weeks	18 months	No
29	24	F	Uveitis	JIA		**Bilateral** – Non-granulomatous uveitis complicated by posteroior synechia, band keratopathy and **bilateral cataract**,** Glaucoma**, Exudative retinal detachment		10 yrs 3 mths	Complete = 2 weeks	4 months	No
30	79	F	Ocular Mucous Membranous Pemphigoid	N/A		**Bilateral** – ocular discomfort, redness, and tearing. Lower eyelid cicatricial entropion with forniceal shortening, symblepharon, tarsal fibrosis, and corneal neovascularization.		29 months	Complete = 8 months	14 months	No
31	70	M	Ocular Mucous Membranous Pemphigoid	N/A		**Right Eye** - Irritation and tearing. Symblepharon of the lower eyelids bilaterally with forniceal shortening and cicatricial entropion of the lower eyelid with trichiasis.		> 42 months	Complete = 8 weeks	12 months	No
32	35	M	Uveitits	Seronegative RA		**Bilateral** – Panuveitis, conjunctival hyperemia and anterior chamber cells, and worsening vitreous opacity, **cataract**, serous retinal detachment		42 months	Clinically Improved = 3 months	31 months	No
33	18	F	Uveitis	N/A		**Bilateral** – Chronic Anterior uveitis: Worsening visual acuity, redness and pain. Mildly hyperemic with retrocorneal deposits. Irregular pupils, adhesions behind the iris OD. pupil membrane closure, pupil light reflection disappeared, posterior iris adhesion, and intraocular lens reign OS, **cataract**		60 months	Clinically Improved = 1 month	10 months	No
34	65	F	Scleritis	N/A		**Left Eye -** redness and severe pain with diminution of vision. deep episcleral congestion, Necrotising and thinning of sclera superiorly, anterior chamber reaction of cells 1 + and flare 1 + with immature senile **cataract**.		25 months	Clinically Improved = 1 month	> 1 month	No
35	24	F	Uveitis	VKH		**Bilateral** – Intermediate and Posterior uveitis, exudative RD. Quiet anterior chamber and plenty of cells in anterior vitreous, and persistent bullous retinal detachment with shifting fluid.		N/A	Clinically Improved = 1 month	1 month	No
36	18	F	Uveitis	JIA		**Bilateral** – Chronic uveitis became chronic with persistent flare in the anterior chamber and development of bilateral synechiae, **cataract**,** band keratopathy**,** glaucoma**		17 years	Clinically improved	5 months	No
37	37	F	Uveitis	JIA		**Bilateral** – Chronic anterior uveitis, + 2 anterior chamber cells, 130 ph/ms anterior chamber flare, **cataract**, band keratopathy		34 years	Clinically Improved	13 months	No
38	21	M	Uveitis	JIA		**Right Eye** – Chronic panuveitis, + 3 anterior chamber cells, 300 ph/ms anterior chamber flare, macular edema, band keratopathy, **cataract**		15 years	Complete = 6 months	4 months	No
39	22	F	Uveitis	JIA		**Bilateral** – AC + 3 cells OD, Band Keratopathy, keratic precipitate, vitiritis, macular edema OU, **cataract**		20 years	Clinically Improved = 9 months	9 months	No
40	45	F	Uveitis	Undefined		**Bilateral** – Pain, photophobia and headache. Anterior uveitis with AC + 2 cells and hypopyon		N/A	Complete = 4 weeks	3 months	No
41	40	F	Scleritis	N/A		**Bilateral** – Ocular pain, redness, swelling and light sensitivity.		N/A	Complete = 1 week	9 months	No
42	59	F	Ulcerative Keratitis	RA		**Right Eye** - Photosensitivity, foreign body sensation, pain, redness, and blurry vision. 2 + conjunctival injection and pericentral ulceration of the cornea with stromal thinning, pannus, and diffuse punctate epithelial erosions.		9 years	Complete = 1 month	1 month	No
43	43	M	Mucous Membrane Pemphigoid	Psoriasis		**Bilateral** - conjunctivitis, subconjunctival fibrosis, symblepharon, corneal neovascularization		8 years	Clinically Improved = 2 months	6 months	No

*JIA Juvenile Idiopathic Arthritis, *RA* Rheumatoid Arthritis, *SLE* Systemic lupus erythematosus, *VKH* Vogt-Kayanagi-Harada Disease, *GPA* Granulomatosis with Polyangiitis

*N/A* Not Available

**Table 5:** Reported use of corticosteroids in different forms (oral, injection, intravenous, topical), initial dosages, and outcomes regarding tapering or discontinuation following initiation of JAK inhibitor therapy

					Corticosteroids Use							
Patient	Age	Gender	Ocular Inflammation	Systematic Diseases		Oral	Ocular Inj.	Intravenous	Topical	Type of CS	Dosage	Tapered down?
1	46	M	Scleritis	N/A		Yes	Yes	No	Yes	Methylprednisolone	64 mg PO OD	N/A
2	28	F	Non-Specific Orbital Inflammation	N/A		Yes	No	Yes	No	Methylprednisolone	64 mg PO OD	Yes
3	38	F	Scleritis	Rheumatoid Arthritis		Yes	Yes	No	Yes	Prednisolone	7.5 mg PO OD	Yes – Discontinued
4	54	F	Scleritis	Rheumatoid Arthritis		Yes	No	No	Yes	Prednisolone	2.5 mg PO OD	Yes – Discontinued
5	61	F	Scleritis	SLE		Yes	No	No	No	Prednisolone	30 mg PO OD	Yes – to 2.5 mg PO OD for SLE
6	28	F	Uveitis	N/A		Yes	No	Yes	No	Methylprednisolone	32 mg PO OD	Yes – Discontinued
7	41	M	Uveitis	N/A		Yes	Yes	Yes	No	Methylprednisolone	12 mg PO OD	Yes – Discontinued
8	59	M	Uveitis	N/A		Yes	No	Yes	No	Methylprednisolone	32 mg PO OD	Yes – to 4 mg QOD
9	42	F	Scleritis	Spondyloarthritis, IgA nephritis		Yes	Yes	No	Yes	N/A	N/A	Yes - Discontinued
10	47	F	Scleritis	GPA		Yes	No	Yes	Yes	Prednisolone	10 mg PO OD	No
11	51	F	Scleritis	N/A		Yes	No	No	Yes	Prednisolone	10 mg PO OD	Yes
12	41	M	Scleritis	N/A		Yes	No	Yes	Yes	Oral Steroid	N/A	Yes - Discontinued
13	41	F	Scleritis	N/A		Yes	No	No	Yes	Oral Steroids	10 mg PO OD	No
14	58	F	Scleritis	N/A		Yes	No	No	Yes	N/A	N/A	Yes - Discontinued
15	39	F	Scleritis	N/A		Yes	No	No	Yes	N/A	N/A	Yes - Discontinued
16	51	M	Scleritis	N/A		Yes	No	No	No	Oral Steroids	20 mg PO OD	Yes – Discontinued
17	35	F	Scleritis	Sjogern’s Syndrome		Yes	No	No	Yes	Oral Steroids	N/A	Yes - Discontinued
18	22	F	Scleritis	N/A		Yes	No	No	Yes	Prednisolone	2.5 mg PO OD	Yes
19	41	M	Scleritis	N/A		Yes	No	Yes	Yes	Oral Steroids	N/A	Yes - Discontinued
20	13	F	Vernal Keratoconjunctivitis	Atopic Dermatitis		No	No	No	Yes	Topical Steroids	N/A	Yes – Discontinued
21	18	F	Vernal Keratoconjunctivitis	Atopic Dermatitis		No	No	No	Yes	Topical Steroids	N/A	N/A
22	41	M	Tattoo granuloma with uveitis (TAGU)	N/A		Yes	No	No	Yes	Prednisolone	30 mg PO OD	Yes – Discontinued
23	53	F	Scleritis & Keratitis	N/A		Yes	No	No	Yes	Prednisolone	1 mg/kg PO OD	Yes – Discontinued
24	18	M	Scleritis	N/A		Yes	No	No	Yes	Prednisolone	40 mg PO OD	Yes – to 5 mg/day
25	47	F	Scleritis	N/A		Yes	No	No	Yes	Prednisolone	1 mg/kg PO OD	Yes – to 2.5 mg/day
26	Adolescent	F	Uveitis	Behcet’s Disease		Yes	Yes	No	No	Oral Steroids	N/A	Yes - Discontinued
27	Thirties	M	Uveitis	Behcet’s Disease		Yes	No	No	No	Prednisolone	60 mg PO OD	N/A
28	85	F	Peripheral Ulcerative Keratitis	RA		Yes	No	Yes	No	Methylprednisolone Prednisolone	250 mg/day for 3 days 30 mg PO OD	Yes Yes – to 2.5 mg/day
29	24	F	Uveitis	JIA		Yes	No	Yes	Yes	Topical Steroids	N/A	Yes
30	79	F	Ocular Mucous Membranous Pemphigoid	N/A		No	No	No	No	No	No	No
31	70	M	Ocular Mucous Membranous Pemphigoid	N/A		No	No	No	No	No	No	No
32	35	M	Uveitits	Seronegative Rheumatoid Arthritis		Yes	No	No	Yes	Prednisolone	60 mg PO OD	No
33	18	F	Uveitis	N/A		Yes	No	No	No	Prednisolone	50 mg PO OD	Yes – to 5 mg/day
34	65	F	Scleritis	N/A		Yes	No	No	Yes	Prednisolone	1 mg/kg	Yes – to 2.5 mg QOD
35	24	F	Uveitis	VKH		Yes	No	Yes	Yes	Methylprednisolone Prednisolone	1 mg IV OD 40 mg PO OD	N/A
36	18	F	Uveitis	JIA		Yes	Yes	No	Yes	Topical Steroids Prednisolone	N/A 12.5 mg PO OD	Yes – Discontinued No – Due to arthritis
37	37	F	Uveitis	JIA		No	Yes	No	No	N/A	N/A	N/A
38	21	M	Uveitis	JIA		Yes	Yes	No	Yes	Prednisolone Topical Steroids	7.5 mg PO OD N/A	No Yes – Discontinued
39	22	F	Uveitis	JIA		No	Yes	No	No	Dexamethasone	700ug implant	N/A
40	45	F	Uveitis	Undefined		Yes	Yes	No	Yes	Prednisolone	80 mg PO BD	Yes – Discontinued
41	40	F	Scleritis	N/A		Yes	Yes	No	Yes	Prednisolone	12 mg PO OD	Yes – Discontinued
42	59	F	Ulcerative Keratitis	Rheumatoid Arthritis		No	No	Yes	Yes	Topical Steroids	Prednisolone acetate 1% TID	Yes – to OD
43	43	M	Mucous Membrane Pemphigoid	Psoriasis		Yes	No	No	No	Prednisolone	6 mg PO OD	No

*JIA Juvenile Idiopathic Arthritis, *RA* Rheumatoid Arthritis, *SLE* Systemic lupus erythematosus, *VKH* Vogt-Kayanagi-Harada Disease, *GPA* Granulomatosis with Polyangiitis

*N/A* Not Available, *OD* once a day, *QOD* Alternative days, *BD *= twice a day

*IV* Intravenous, *PO* Oral

JAK inhibitors demonstrated sustained efficacy in specific conditions, including scleritis (84% complete remission), uveitis (64%), vernal keratoconjunctivitis with atopic dermatitis (both cases achieving complete resolution), and ocular mucous membrane pemphigoid (two of three cases achieving complete remission).

The pooled data suggest a favorable safety profile for JAK inhibitors. Adverse events were mild and included elevated liver transaminases (*n* = 2; baricitinib), herpes virus reactivation (*n* = 1; tofacitinib), mild leukopenia and transaminitis (*n* = 1; upadacitinib), and neutropenia (*n* = 1; baricitinib).

Regarding quality assessment, all 22 articles were evaluated using the Murad et al. tool, which assesses four domains: selection, ascertainment, causality, and reporting (Table 6) [7]. In the selection domain, only three articles explicitly reported the entire experience of the center and authors, ensuring that no similar presentations were excluded. For ascertainment, all articles adequately documented both exposure and outcome, apart from Baquet-Walscheid et al., where the exposure was unclear. Ten articles described investigations undertaken to rule out alternative diagnoses, while the many of the remaining studies primarily reported cases in which diagnoses had already been established prior to presentation.

**Table 6:** – Quality assessment of the 22 included articles using the Murad et al. tool, which evaluates eight items across four domains: selection, ascertainment, causality, and reporting

CASE REPORTS/SERIES	Q1 - Does the patient(s) represent(s) the whole experience of the center?	Q2 - Was the exposure adequately ascertained?	Q3 - Was the outcome adequately ascertained?	Q4 - Were other alternative causes that may explain the observation ruled out?	Q5 - Was there a challenge/re-challenge phenomenon?	Q6 - Was there a dose–response effect?	Q7 - Was follow-up long enough for outcomes to occur?	Q8 - Is the case(s) described with sufficient details?
Vidic Krhlikar et al. (2024)	No	Yes	Yes	Yes	No	No	Yes	Yes
Chung Young Kim et al. (2025)	No	Yes	Yes	No	No	No	Yes	Yes
Vidic Krhlikar et al. (2025)	Yes	Yes	Yes	Yes	No	No	Yes	Yes
Baquet-Walscheid et al. (2022)	No	No	Yes	No	No	No	Yes	No
Pyare et al. (2024)	Yes	Yes	Yes	Yes	No	No	Yes	Yes
Kito et al. (2024)	No	Yes	Yes	No	No	No	Yes	No
Mimo et al. (2024)	No	Yes	Yes	No	No	No	Yes	Yes
Yang et al. (2024)	No	Yes	Yes	Yes	No	No	Yes	Yes
Dey et al. (2023)	No	Yes	Yes	Yes	No	No	Yes	No
Tao et al. (2023)	No	Yes	Yes	No	No	No	Yes	No
Calvo-Río et al. (2022)	No	Yes	Yes	No	No	No	Yes	Yes
Baquet-Walscheid et al. (2022)	No	Yes	Yes	No	No	No	Yes	Yes
James et al. (2021)	No	Yes	Yes	No	No	No	Yes	Yes
Kaneko et al. (2021)	No	Yes	Yes	No	No	No	Yes	Yes
Liu et al. (2021)	No	Yes	Yes	Yes	No	No	Yes	Yes
Pyare et al. (2020)	No	Yes	Yes	Yes	No	No	No	No
Majumder et al. (2020)	No	Yes	Yes	No	No	No	No	No
Miserocchi et al. (2020)	Yes	Yes	Yes	Yes	No	No	Yes	Yes
Bauermann et al. (2019)	No	Yes	Yes	Yes	No	No	Yes	No
Paley et al. (2019)	No	Yes	Yes	Yes	No	No	Yes	No
Sarny (2018)	No	Yes	Yes	No	No	No	Yes	Yes
Meadow et al. (2014)	No	Yes	Yes	No	No	No	No	No

Regarding causality, none of the included articles discussed a clear challenge–rechallenge phenomenon or a dose–response relationship. Follow-up duration was sufficient to assess the efficacy and safety of treatment in most cases, although three studies reported follow-up periods of one month or less, which was insufficient for a thorough evaluation. Finally, in the reporting domain, while only a subset of cases provided highly detailed narratives, all studies contained adequate information to meet inclusion criteria for this review.

## Discussion

JAK inhibitors represent a promising class of immunomodulatory agents that act by inhibiting the JAK/STAT signaling pathway, thereby downregulating pro-inflammatory gene expression. These “targeted synthetic DMARDs” have been extensively studied in conditions such as rheumatoid arthritis and ulcerative colitis [8, 9]. Several randomized controlled trials have demonstrated that JAK inhibitor monotherapy, such as upadacitinib and tofacitinib, is superior to cDMARDs, particularly methotrexate, in achieving disease remission in rheumatoid arthritis [10, 11]. Furthermore, JAK inhibitors have also been shown to outperform adalimumab in regarding to the degree of clinical improvement in rheumatoid arthritis [12]. The robust efficacy of JAK inhibitors in systemic autoimmune diseases, along with the evidence summarized in this review, highlights their potential to replace or complement cDMARDs and bDMARDs in autoimmune ocular inflammatory diseases.

This systematic review objective was a synthesis of the evidence supporting the use of JAK inhibitors in autoimmune ocular inflammatory diseases. Although several clinical trials have been initiated to investigate this therapeutic class in ocular inflammation, none have published definitive results to date. The HUMBOLDT trial, for instance, which evaluated the efficacy and safety of filgotinib in non-infectious uveitis, reported a reduced rate of treatment failure with filgotinib compared to placebo; however, the study was terminated prematurely for financial reasons before reaching its primary endpoint [13]. Additionally, the JUVE-BRIGHT trial, designed to assess baricitinib in juvenile idiopathic arthritis–associated uveitis or chronic anterior antinuclear antibody–positive uveitis, has yet to report its results [14]. 

One notable observation from our review is that two cases of uveitis required a switch in their JAK inhibitor regimen from baricitinib and tofacitinib to upadacitinib. The former agents are classified as non-selective JAK inhibitors, targeting JAK1, JAK2, and, in the case of tofacitinib, JAK3 as well. In contrast, upadacitinib selectively inhibits JAK1, which regulates several key pro-inflammatory cytokines, including IL-2, IL-6, IL-12, IL-23, and interferons (IFN-α, IFN-β, IFN-γ). An animal model study demonstrated upregulation of JAK1-mediated signaling cascades in autoimmune uveitis, supporting the therapeutic potential of selective JAK1 inhibitors in this context [15]. Additionally, reports suggests that selective JAK inhibitors may be associated with a lower incidence of adverse effects compared to non-selective agents [16]. In our review, among eight patients treated with selective JAK inhibitors (upadacitinib and abrocitinib), only one experienced mild adverse events (elevated liver transaminases and leukopenia). In contrast, 14.2% of patients receiving non-selective JAK inhibitors reported adverse effects, which suggests a favorable adverse-event profile of selective JAK1 inhibition.

In our review, JAK inhibitors demonstrated comparable effectiveness as monotherapy or in combination with cDMARDs or bDMARDs, with no clear differences in time to remission, adverse events, or risk of relapse. A systematic review in rheumatoid arthritis involving 2,290 patients reported that combination therapy with JAK inhibitors and methotrexate achieved higher rates of clinical remission than JAK inhibitor monotherapy, albeit at the cost of increased adverse events [17]. 

Adverse events observed in our review were generally mild, ranging from herpetic reactivation to elevated transaminases, and no life-threatening complications were reported among the 43 patients. However, these findings should be interpreted cautiously, as larger drug-safety databases have documented a broader spectrum of ocular and systemic adverse effects. For instance, a European database analysis of over 1,400 patients treated with baricitinib or tofacitinib reported ophthalmic adverse events, including paradoxical scleritis, uveitis, cataract, and retinal vascular thrombosis, with a mean time to onset of 127 days for baricitinib and 360 days for tofacitinib [18]. 

An important outcome highlighted in our review was the potential of JAK inhibitors to facilitate steroid tapering. Long-term corticosteroid use is associated with well-documented ocular complications, including cataract, glaucoma, and increased susceptibility to infections [19]. Approximately 75% of the patients in our review were able to reduce or discontinue steroids following initiation of JAK inhibitors. Findings from a prospective study in rheumatoid arthritis revealed that over 30% of patients treated with tofacitinib successfully tapered steroids within 12 weeks [20]. In our cohort, 40% of patients initially treated with steroids were able to discontinue them completely, which is higher than reported. These results support the role of steroids as bridging therapy during induction of remission, followed by tapering once disease control is achieved to minimize steroid-related morbidity.

This review has several limitations. As it is based solely on case reports and series, the findings are subject to significant heterogeneity and reporting bias, limiting the ability to generalize results. Selection bias is also likely, as clinicians may preferentially report positive outcomes, potentially inflating observed efficacy rates. These limitations underscore the need for larger, prospective, controlled studies enrolling patients with homogeneous clinical profiles to assess the true efficacy and safety of JAK inhibitors more accurately in autoimmune ocular inflammatory diseases. Comprehensive reporting of both positive and negative outcomes is critical to building a balanced evidence base.

In conclusion, JAK inhibitors may be an effective and relatively safe therapeutic option for patients with autoimmune ocular inflammatory diseases, offering high rates of remission, low relapse rates, and the ability to taper steroids, thereby reducing steroid-associated complications. Further larger, controlled studies are needed to better define their role and long-term safety in this setting.

## Data Availability

The author confirms that all data generated or analysed during this study are included in this published article.
